# High risk exposure to HIV among sexually active individuals who tested negative on rapid HIV Tests in the Tshwane District of South Africa—The importance of behavioural prevention measures

**DOI:** 10.1371/journal.pone.0192357

**Published:** 2018-02-02

**Authors:** Simnikiwe H. Mayaphi, Desmond J. Martin, Steve A. S. Olorunju, Brian G. Williams, Thomas C. Quinn, Anton C. Stoltz

**Affiliations:** 1 Department of Medical Virology, University of Pretoria, City of Tshwane, South Africa; 2 National Health Laboratory Service-Tshwane Academic Division (NHLS-TAD), City of Tshwane, South Africa; 3 Toga Laboratories, Johannesburg, South Africa; 4 Biostatistics unit, Medical Research Council, City of Tshwane, South Africa; 5 South African Centre for Epidemiological Modelling and Analysis, Stellenbosch, South Africa; 6 Division of Intramural Research, National Institute of Allergy and Infectious Diseases, National Institutes of Health, Bethesda, Maryland, United States of America; 7 Johns Hopkins University School of Medicine, Baltimore, Maryland, United States of America; 8 Division of Infectious Diseases, Department of Internal Medicine, University of Pretoria, City of Tshwane, South Africa; University of Toronto, CANADA

## Abstract

**Objective:**

To assess the prevalence of HIV risk behaviour among sexually active HIV sero-negative individuals in the Tshwane district of South Africa (SA).

**Methods:**

Demographic and HIV risk behaviour data were collected on a questionnaire from participants of a cross-sectional study that screened for early HIV infection using pooled nucleic acid amplification testing (NAAT). The study enrolled individuals who tested negative on rapid HIV tests performed at five HIV counseling and testing (HCT) clinics, which included four antenatal clinics and one general HCT clinic.

**Results:**

The study enrolled 9547 predominantly black participants (96.6%) with a median age of 27 years (interquartile range [IQR]: 23–31). There were 1661 non-pregnant and 7886 pregnant participants largely enrolled from the general and antenatal HCT clinics, respectively. NAAT detected HIV infection in 61 participants (0.6%; 95% confidence interval [CI]: 0.4–0.8) in the whole study. A high proportion of study participants, 62.8% and 63.0%, were unaware of their partner’s HIV status; and also had high prevalence, 88.5% and 99.5%, of recent unprotected sex in the general and pregnant population, respectively. Consistent use of condoms was associated with protection against HIV infection in the general population. Trends of higher odds for HIV infection were observed with most demographic and HIV risk factors at univariate analysis, however, multivariate analysis did not show statistical significance for almost all these factors. A significantly lower risk of HIV infection was observed in circumcised men (*p* <0.001).

**Conclusions:**

These data show that a large segment of sexually active people in the Tshwane district of SA have high risk exposure to HIV. The detection of newly diagnosed HIV infections in all study clinics reflects a wide distribution of individuals who are capable of sustaining HIV transmission in the setting where HIV risk behaviour is highly prevalent. A questionnaire that captures HIV risk behaviour would be useful during HIV counselling and testing to ensure that there is a systematic way of identifying HIV risk factors and that counselling is optimised for each individual. HIV risk behaviour surveillance could be used to inform relevant HIV prevention interventions that could be implemented at a community or population level.

## Introduction

Human immunodeficiency virus (HIV) is a global health problem that has disproportionately affected sub-Saharan Africa [1]. In this region, the highest burden of HIV infections is found in South Africa (SA) where there are about 6.19 million people living with HIV [2]. Thus, SA contributes approximately 20% of HIV infections to the global HIV pandemic [1] despite having about 1% of the world’s population [3].

HIV is transmitted through sexual intercourse, blood or blood products and by vertical transmission. Sexual transmission plays a big role in HIV transmission contributing to about 80% of transmissions or more [4]. Unprotected sex is the major determinant for sexual acquisition and transmission of HIV [5, 6]. The presence of high HIV viral load (VL) of at least 1500 copies in an individual infected with HIV is associated with a risk of sexual transmission to uninfected individuals [7]. Various other factors play a significant role in genital acquisition of HIV during unprotected sex, such as the presence of other sexually transmitted infections (STIs) [8] and male circumcision status [6]. Failure to respond to antiretroviral (ARV) therapy creates opportunities for transmission of ARV drug resistant strains of HIV [9].

Preventing new infections is important for controlling the HIV pandemic. Strategies for preventing HIV infections include behavioural, biomedical and structural approaches. Behavioural prevention measures aim at reducing people’s HIV risk behaviour, and form an integral part of any HIV prevention approach as they are also needed for risk management in individuals infected with HIV in order to avoid further spread of HIV. Successes of HIV prevention have been achieved with behavioural measures in some countries, however, these successes were often short-lived and difficult to maintain over a long time [10]. For instance, in early to mid-1990s a decline of HIV prevalence and incidence was observed in Uganda and Thailand owing to behavioural prevention measures [11–13]. However, this was later followed by increasing trends of HIV prevalence and incidence in the general population and in some key population groups [14–17]. The other challenge of the behavioural prevention measures is that their success could not be reproduced in some parts of the world [10].

The world continues to see a high rate of new HIV infections despite the availability of prevention measures [1]. Of all the HIV prevention measures, the behavioural measures are the most affordable and easier to implement even in low resource settings. Therefore, it is important to continually make efforts to understand and improve behavioural prevention measures against HIV. This study assessed HIV risk behaviours among sexually active HIV sero-negative people attending HIV counselling and testing (HCT) clinics in the Tshwane district of SA.

## Methods

Participants were enrolled as part of a study that screened for early HIV infection from 5 HCT clinics in the Tshwane region of SA. Four of these clinics were antenatal clinics and one was a general HCT clinic. The inclusion criteria for this study were a negative rapid HIV test and recent sexual activity as defined by having had sex within the last 3 months. The pregnant females were enrolled during their first antenatal care visit just after they had done the HIV test. Pooled nucleic acid amplification testing (NAAT) was performed in mini-pools of 5 samples using the Roche CAP/CTM HIV VL version 2 assay (Roche Diagnostics, Mannheim, Germany), followed by individual sample testing in positive pools. NAAT-positive participants were recalled to the clinics for confirmatory testing and appropriate management. Data on serological characterisation of NAAT-positive individuals have previously been published [18].

At enrolment data on demographics and HIV risk behaviour were collected using a newly designed questionnaire, which captured information on; age, race, marital status, condom use, HIV status of the partner, current number of sexual partners, alcohol and drug use, commercial sex work by females, recent history of STIs (within the last 3 months), male circumcision, sex with other men, recent history of sexual assault (within the last month), history of travel to other countries, and recent flu-like illness (S1 Fig). The latter was not included in the analysis as it is not a component of risk behaviour but a manifestation of HIV infection.

Questionnaires were in English and administered by research assistants who had received training in HCT and had been providing HCT at the clinics prior to their involvement in this study. Questionnaire was administered to one participant at a time and this process took about 5 minutes to complete. This was done in a private space, mostly in the counseling room after the prospective participants had just received their negative rapid HIV test results. All the research assistants knew the commonly used languages in SA; hence it was easier that they administer the questionnaires. Participants were given an option of not answering questions that they were not comfortable with. The questionnaire data were then captured on an Excel spreadsheet using codes to simplify capturing. Quality assurance of data capturing included training all the research assistants who captured data into Excel, verification of correct codes in each column of Excel spreadsheet, and spot check verification comparing the original questionnaire documents to what was captured. Validation of the questionnaire tool was done in a subset of participants using a follow up questionnaire at a different time point. A few questions from the original questionnaire were not included in the validation questionnaire as data for certain parameters would have changed with time (S2 Fig).

The study was approved by the University of Pretoria’s Faculty of Health Sciences Ethics Committee (Protocol number–295/2015) and by Tshwane Research Ethics Committee (TMREC 2010/26). The legal ages for consenting to HIV testing and medical treatment in SA are 12 and 14 years, respectively [19]. This study enrolled participants aged 14 years or older. The initial study protocol was approved for participants older than 18 years but it was later amended and approved to include those from 14 years of age to extend the benefits of screening for early HIV infection to the younger age groups, most of whom came to the clinics alone. All study participants agreed to participate and signed written consent forms before enrolment.

### Statistical analysis

A descriptive analysis was used to present summary statistics (median, proportions and 95% confidence interval [CI]) for the parameters. Stratification of study participants was done according to general and antenatal HCT clinics, and then further stratification was done in each population group according to demographic and HIV risk factors. Univariate logistic regression was done on stratified data in order to identify factors that are associated with a risk of HIV infection. Distribution of newly identified HIV infections in each parameter was used to assess association with the risk of HIV. All factors associated with the risk of HIV infection in the univariate analysis, with *p*-value of 0.2 or less, were included in the multivariate logistic regression analysis. Questionnaire parameters that were not applicable to the whole general or pregnant population group were not included in the analysis, but a few of these were analysed separately such as data for males. Comparison of the proportion of HIV infections for data on circumcision and men who have sex with men was done using two sample t-test for proportions. Missing data occurred at a low rate (≤2%) for most parameters and were excluded from the analysis. The only parameter with a high rate of missing data was the “number of current sexual partners” owing to amendment of the initial questionnaire, which assessed “number of lifetime sexual partners.” This amendment was done early in the study after enrolling about 300 participants from the general HCT clinic, where the study was initially conducted. Missing data for this parameter were also excluded from the analysis. All the statistics were performed on the STATA version 14.2 software (StataCorp LP, College Station, TX, USA).

## Results

### Demographics

From March 2012 to June 2016, the study enrolled 9547 predominantly black participants (96.6%). Their median age was 27 years (interquartile range [IQR]: 23–31). There were 1661 non-pregnant participants largely enrolled from the general HCT clinic, while there were 7886 pregnant females largely enrolled from the antenatal clinics. Majority of the non-pregnant (general) population were males (54.7%). Similar trends of demographic and HIV risk factors were observed between the two population groups. For instance, most participants from both population groups were unmarried, had reported inconsistent use or no use of condoms, and had no knowledge of their partner’s HIV status (Table 1). A significant proportion of participants with consistent condom use reported having had a recent condom burst. Few participants reported having multiple current sexual partners, recent STIs, sexual assault, alcohol or other drugs consumption, and frequent travel. Those who reported use of other drugs only mentioned use of marijuana. Most men (99%) were heterosexual men and 54.3% said they were circumcised (Table 1). None of the study participants reported involvement in commercial sex work. Questionnaire validation was done in a small subset of pregnant participants (*n* = 133). The questionnaire data that was obtained at a different time point for validation purposes showed similar trends as the data collected at enrolment, indicating that the information obtained on the original questionnaire was reliable (S1 Table).

**Table 1:** Demographic and behavioural risk factors of all study participants.

FACTORS	SUB-GROUPS	GENERAL POPULATION % (n = 1661)	PREGNANT POPULATION % (n = 7886)
**Age**	14–24 years	21.7 (361)	38.1 (3007)
	25–49 years	72.7 (1208)	61.9 (4879)
	50+ years	5.5 (92)	(0)
**Gender**	Males	54.7 (908)	n/a
	Females	45.3 (753)	100 (7886)
**Marital status**	Unmarried	72.5 (1205)	69.5 (5479)
	Married	27.4 (455)	30.4 (2400)
	No data	0.1 (1)	0.1 (7)
**Condom use**	Consistent*	11.4 (190)	0.5 (42)
	Inconsistent	43.1 (716)	51.9 (4092)
	No use	45.4 (754)	47.5 (3742)
	No data	0.1 (1)	0.1 (10)
**Partner’s HIV status**	Positive	12.1 (201)	0.2 (14)
	Negative	25.0 (416)	36.7 (2898)
	Unknown	62.8 (1043)	63.0 (4968)
	No data	0.1 (1)	0.1 (6)
**Number of current sexual partners**	One	72.5 (1204)	99.6 (7855)
	Multiple	9.7 (162)	0.3 (22)
	No data	17.8 (295)	0.1 (9)
**Recent STI** (self-reported)	Yes	6.3 (104)	3.0 (240)
	No	93.6 (1555)	96.8 (7637)
	No data	0.1 (2)	0.1 (9)
**Recent sexual assault**	Yes	0.2 (3)	0.1 (8)
	No	99.7 (1656)	99.7 (7860)
	No data	0.1 (2)	0.2 (18)
**Alcohol use**	Yes	46.3 (770)	4.7 (373)
	No	53.6 (890)	95.2 (7506)
	No data	0.1 (1)	0.1 (7)
**Other drugs**	Yes^†^	1.4 (23)	(0)
	No	98.5 (1637)	99.9 (7876)
	No data	0.1 (1)	0.1 (10)
**Frequent travel to other countries**	Yes	6.6 (110)	2.0 (161)
	No	93.3 (1549)	97.8 (7716)
	No data	0.1 (2)	0.1 (9)
**Male circumcision**	Yes	54.3 (487)	n/a
	No	45.7 (410)	n/a
	No data	1.2 (11)	n/a
**Sex with other men**	Yes	1 (9)	n/a
	No	99 (881)	n/a
	No data	2.0 (18)	n/a

* = condom burst was reported by 52.1% (n = 99) of participants in the general population and by 90.5% (n = 38) in the pregnant population. STI = sexually transmitted infection, < = less than,

^**†**^ = only marijuana was reported, n/a- not applicable.

### Newly diagnosed HIV infections

Of all the study participants (i.e. with negative rapid tests at enrolment), 61 tested positive on NAAT, resulting in 0.6% (95% CI: 0.4–0.8) of newly diagnosed HIV infections. These infections were identified in all 5 study clinics with variable prevalence, ranging from 0.3% to 1.2%. Serological characterisation was performed and data for the first 55 participants with newly diagnosed HIV infection have been published previously. Participants with newly diagnosed HIV infection consisted of individuals with either early HIV infection or chronic HIV infection, all of whom were missed by rapid tests at the points-of-care [18]. The median VL of participants with newly diagnosed infections was 17536 copies/ml (IQR: 4877–85037), and these participants also had other risk factors that enhance secondary transmission of HIV (S2 Table).

### Risk factors and association with HIV infection

Trends of higher odds for HIV infection were observed with some demographic and HIV risk factors at univariate analysis (Table 2). However, multivariate analysis did not show statistical significance for all these factors except for recent sexual assault (p = 0.001, CI: 5.22–865.53) in the general population, but this was a weak finding as there were very few participants who reported recent sexual assault (Table 3).

**Table 2:** Univariate analysis of factors associated with HIV infection.

		GENERAL POPULATION			PREGNANT POPULATION		
		HIV INFECTIONS	UNIVARIATE ANALYSIS		HIV INFECTIONS	UNIVARIATE ANALYSIS	
FACTORS	SUB-GROUPS	% (*n* / sub-group)	ODDS ratio (95% CI)	P-value	% (*n* / sub-group)	ODDS ratio (95% CI)	P-value
**Age**	14–24 years	0.8 (3/361)	1		0.5 (14/3007)	1	
	25–49 years	0.8 (10/1208)	1.0 (0.27–3.64)	0.995	0.7 (34/4879)	1.5 (0.80–2.80)	0.203
	50+ years	-- (0/92)	--		--		
**Gender**	Males	0.6 (5/908)	0.52 (0.18–1.58)	0.247	--		
	Females	1.1 (8/753)	1		0.6 (48/7886)	--	
**Marital status**	Unmarried	0.9 (11/1205)	2.09 (0.46–9.45)	0.340	0.7 (39/5479)	1.90 (0.92–3.94)	0.082
	Married	0.4 (2/455)	1		0.4 (9/2400)	1	
**Condom use**	Consistent	-- (0/190)	--		2.3 (1/43)*	5.05 (0.66–38.69)	0.119
	Inconsistent	0.8 (6/716)	1		0.7 (29/4091)	1.48 (0.82–2.66)	0.195
	No use	0.9 (7/754)	1.11 (0.37–3.31)	0.853	0.5 (18/3742)	1	
**Partner’s HIV status**	Positive	1.5 (3/201)	1.96 (0.51–7.45)	0.553	-- (0/14)	--	
	Negative	0.5 (2/416)	0.63 (0.13–2.96)	0.323	0.4 (13/2898)	0.63 (0.33–1.20)	0.163
	Unknown	0.8 (8/1043)	1		0.7 (35/4968)	1	
**Number of current sexual partners**	One	0.8 (10/1204)	1		0.6 (48/7855)	--	
	Multiple	0.6 (1/162)	0.82 (0.10–6.55)	0.855	-- (0/22)	--	
**Recent STI** (self-reported)	Yes	1.9 (2/104)	2.75 (0.60–12.58)	0.192	0.4 (1/240)	0.68 (0.93–4.92)	0.699
	No	0.7 (11/1555)	1		0.6 (47/7637)	1	
**Recent sexual assault**	Yes	33.3 (1/3)	68.5 (5.81–807.18)	0.001	-- (0/8)	--	
	No	0.7 (12/1656)	1		0.6 (48/7860)	--	
**Alcohol use**	Yes	0.6 (5/770)	0.72 (0.23–2.21)	0.567	0.5 (2/373)	0.87 (0.21–3.61)	0.853
	No	0.9 (8/890)	1		0.6 (46/7506)	1	
**Other drugs**	Yes^†^	4.3 (1/23)	6.16 (0.77–49.41)	0.087	-- (0)	--	
	No	0.7 (12/1637)	1		0.6 (48/7876)	--	
**Frequent travel to other countries**	Yes	1.8 (2/110)	2.59 (0.57–11.83)	0.220	-- (0/161)	--	
	No	0.7 (11/1549)	1		0.6 (48/7716)	--	

CI = confidence interval, -- = not applicable, STI—sexually transmitted infection,

* = the infected participant in this sub-group had reported condom burst,

^**†**^ = only marijuana was reported. Data on male circumcision and men who have sex with men were analysed separately.

**Table 3:** Multivariate analysis of factors associated with HIV infection.

		GENERAL POPULATION			PREGNANT POPULATION		
		HIV INFECTIONS	MULTIVARIATE ANALYSIS		HIV INFECTIONS	MULTIVARIATE ANALYSIS	
FACTORS	SUB-GROUPS	% (*n* / sub-group)	ODDS ratio (95% CI)	P-value	% (*n* / sub-group)	ODDS ratio (95% CI)	P-value
**Age**	14–24 years	0.8 (3/361)	--		0.5 (14/3007)	1	
	25–49 years	0.8 (10/1208)	--		0.7 (34/4879)	1.82 (0.96–3.45)	0.066
	50+ years	-- (0/92)	--		--		
**Gender**	Males	0.6 (5/908)	--		--		
	Females	1.1 (8/753)	--		0.6 (48/7886)	--	
**Marital status**	Unmarried	0.9 (11/1205)	--		0.7 (39/5479)	2.00 (0.89–4.49)	0.091
	Married	0.4 (2/455)	--		0.4 (9/2400)	1	
**Condom use**	Consistent	-- (0/190)	--		2.3 (1/43)*	5.02 (0.63–39.66)	0.126
	Inconsistent	0.8 (6/716)	--		0.7 (29/4091)	1.15 (0.60–2.22)	0.674
	No use	0.9 (7/754)	--		0.5 (18/3742)	1	
**Partner’s HIV status**	Positive	1.5 (3/201)	--		-- (0/14)	--	
	Negative	0.5 (2/416)	--		0.4 (13/2898)	0.65 (0.34–1.25)	0.198
	Unknown	0.8 (8/1043)	--		0.7 (35/4968)	1	
**Number of current sexual partners**	One	0.8 (10/1204)	--		0.6 (48/7855)	--	
	Multiple	0.6 (1/162)	--		-- (0/22)	--	
**Recent STI** (self-reported)	Yes	1.9 (2/104)	2.07 (0.41–10.52)	0.382	0.4 (1/240)	--	
	No	0.7 (11/1555)	1		0.6 (47/7637)	--	
**Recent sexual assault**	Yes	33.3 (1/3)	67.2 (5.22–865.53)	0.001	-- (0/8)	--	
	No	0.7 (12/1656)	1		0.6 (48/7860)	--	
**Alcohol use**	Yes	0.6 (5/770)	--		0.5 (2/373)	--	
	No	0.9 (8/890)	--		0.6 (46/7506)	--	
**Other drugs**	Yes^†^	4.3 (1/23)	6.15 (0.74–51.07)	0.093	-- (0)	--	
	No	0.7 (12/1637)	1		0.6 (48/7876)	--	
**Frequent travel to other countries**	Yes	1.8 (2/110)	2.69 (0.57–12.62)	0.211	-- (0/161)	--	
	No	0.7 (11/1549)	1		0.6 (48/7716)	--	

CI = confidence interval, -- = not applicable, STI—sexually transmitted infection,

* = the infected participant in this sub-group had reported condom burst,

^**†**^ = only marijuana was reported.

Interestingly, no HIV infection was found in participants who reported consistent condom use in the general population despite a high rate of condom burst. The single participant who reported consistent condom use in the pregnant population and found to have HIV infection belonged to a sub-group of participants who reported recent condom burst (Table 2). A significantly lower risk of HIV infection was observed in circumcised men (*p* <0.001). A small proportion (1%) of men reported having sex with other men but no HIV infection was found among them.

### Other HIV risk behaviours

There was a very high prevalence of recent unprotected sex among study participants as evidenced by the high rate of inconsistent use or no use of condoms and high rate of condom burst in those who reported consistent condom use (Table 1). HIV infections were also identified in some married participants and in the group of participants that reported to have HIV-negative partners (Table 2). Among the participants with newly diagnosed HIV infection who reported having HIV-positive partners, there was one whose partner was already on ARV therapy. One male participant (#7, Pt ID 5041) with concurrent sexual partners who was diagnosed with new HIV infection had four sexual partners and a very high HIV viral load of 22 million copies/mL (S2 Table).

## Discussion

### Demographics and HIV infections

This study assessed the prevalence of HIV risk behaviour among sexually active HIV sero-negative individuals who were screened for early HIV infection in the Tshwane district of SA. The finding of 0.6% newly diagnosed HIV infections in the whole study population highlights the problem of ongoing transmission of HIV despite the availability of many HIV prevention measures [1]. A higher prevalence of these infections in females compared to males in the general population (Table 2) was not surprising as HIV prevalence is generally higher in females [20]. The newly diagnosed HIV-infected participants were found in all the study clinics, highlighting a wide distribution of individuals who are capable of sustaining the HIV transmission in the setting where there is a high prevalence of unprotected sex (Table 1). It is not surprising that HIV infections were only observed in participants aged 14–49 years in this study (Table 2). This is consistent with other data that have showed a significantly higher incidence of HIV in sexually active people within this age group [20].

The trend of higher risk of HIV infection among unmarried individuals compared to the married ones (Table 2) has been observed in a previous South African study [21]. The finding of HIV infections among married participants has also been observed by many other studies in the sub-Saharan Africa [21–24]. Extramarital affairs could be the possible source of HIV infection in married couples [23]. Another possible explanation is if one partner was in the window period if the couple did HIV testing prior to marriage [18]. Individuals who are in sero-discordant relationships are at an increased risk of HIV acquisition especially when the VL of the infected partner is ≥1500 copies/mL [7]. The majority of participants with newly diagnosed HIV infection had VL above this threshold (S2 Table) showing a higher likelihood of secondary spread of HIV infection. It was not surprising to find that circumcision was associated with a significantly reduced risk of HIV infection as it has been reported to offer protection against HIV acquisition [6, 25].

### Condom use and HIV infections

The absence of HIV infection among participants who reported consistent condom use in the general population (Table 2) highlights the protective effect associated with consistent condom use. The high rate of condom burst reduces the protective effect of consistent condom use as observed in the pregnant population where a single HIV infection was found in the sub-group that reported consistent condom use. This highlights that a proper and consistent use of condoms is important for protective effect of condoms against HIV acquisition. When condoms are used properly, their effectiveness could be as high as 95% in preventing acquisition of HIV [26, 27]. Our study shows that consistency of condom use and recent history of condom burst should be measured by future HIV studies or surveys that look at the protective effect of condoms rather than measuring condom use at last sex. Attempts of reducing condom burst should be made in order to maximise the benefits of consistent condom use.

The risk of HIV infection between the group of participants with inconsistent condom use and the other with no condom use looked similar. This indicates that people with inconsistent condom use are as exposed to HIV infection as those who do not use condoms. Inconsistent use of condoms has been associated with high risk of acquiring HIV infection [26, 28]. Most HIV studies measure condom use at last sex as the proxy for protected sex [20, 29]. This may not be a good practice as people with inconsistent condom use may report having used condoms at last sex, while they were exposed to HIV at the times they did not use condoms. Some researchers have reported impressive rates of last sex condom use [20], but this did not seem to translate to protection against HIV infection as the rate of new HIV infections remains high globally and in high endemic areas such as SA [1, 20].

### Partner’s HIV status and HIV infections

The finding that majority of the study participants in both population groups did not know their partner’s HIV status (Table 1) reflects the observation from other studies that most people are unaware of their HIV status and/or that of their sexual partners [24, 30]. This reflects on low rate of couples’ HIV testing, which has been reported for most countries in the sub-Saharan Africa [24]. Couples’ HCT is an effective way of counselling as it ensures that both partners know their HIV status, and thus reduces the risk of HIV transmission [22, 24, 31]. Our study data highlight a need to promote couple’s HCT in South Africa. Newly diagnosed HIV infections were also identified among participants who reported that their partner’s HIV status was negative (Table 2). The reason for this could be that their partners were in the window period or had false-negative HIV test results the last time they tested for HIV [18], or that they lied about their HIV status. The diagnosis of HIV infection in a participant whose partner was already on ARV therapy indicates a risk for acquisition of HIV drug resistant virus. South Africa has the biggest ARV programme in the world [32], and has a substantial number of patients who have failed first line regimen [33]. This puts individuals who are exposed to HIV at high risk of acquiring HIV drug-resistant mutants, which compromise response to ARV therapy. Transmission of HIV drug-resistant strains has been reported in SA and other sub-Saharan African countries [9, 34].

### Number of current sexual partners and HIV infections

A low rate of concurrent sexual partnerships was observed in this study (Table 1). The diagnosis of HIV infection in a male participant who reported having four concurrent sexual partners and found to have a very high HIV viral load of 22 million copies/ml (S2 Table) highlights the potential of concurrent sexual partnerships to exponentially increase the number of individuals infected with HIV. Concurrent sexual relationships play a significant role in HIV transmission and epidemiology even though they occur at a lower rate in some settings. A study conducted in Botswana observed a similar trend of a lower rate of concurrent sexual partnerships compared to monogamous partnerships [35]. Some of the participants with newly diagnosed HIV infection reported having changed the sexual partners by the time they came back for follow up. This observation was also made with some HIV-uninfected individuals who were included in the questionnaire validation arm of the study. This could be an indication that although most people are in monogamous relationships, there is a substantially higher rate of sexual partner exchange or serial monogamous relationships, which favours transmission of HIV. Both serial monogamous relationships and concurrent sexual relationships have been implicated in driving the HIV epidemiology in sub-Saharan Africa [36, 37].

### Trends on multiple behaviours and HIV infections

Only a small proportion of participants reported sexual assault, and this was associated with a significantly higher risk of HIV infection in the general population. This is probably caused by the fact that abused individuals are not provided with options for safe sex [38]. Even though there was lack of significant association with HIV infection found with other risk factors such as STIs, travel history, and use of alcohol or other drugs use; there are published data that have shown a higher risk of HIV infection associated with these factors [8, 39, 40]. It is not uncommon to observe non-significant trends of HIV association with risk factors in behavioural studies. The complexity of understanding the specific behaviours that significantly contribute to HIV acquisition has been observed in other studies. These studies, like ours, failed to show statistically significant findings despite observing trends associating HIV infection with certain behavioural risk factors [10, 21, 36, 37]. Coates et al summed up this phenomenon by saying–“failure to show that a specific strategy reduces HIV infections does not render it useless in a comprehensive programme or a multilevel behavioural strategy for HIV prevention [10].” HIV risk behaviour studies probably suffer from the dynamics of a relationship, where a risk is determined by the total couple’s behaviour. Thus, enrolling into a study the partner with less behavioural risk(s) who is infected with HIV due to the other partner’s behaviour could easily mislead the interpretation of study data. This is reflected in certain parameters in this study, such as identification of HIV infections in married participants and in those who reported that their partners were HIV-negative (Table 2).

### HIV risk assessment during counselling

Currently, there is no tool used for assessing or capturing HIV risk data during counselling in most South African HCT clinics. Thus, it is difficult to know if all counsellors assess HIV risk factors and address such factors during counselling for each and every individual. A study that assessed the quality of HIV counselling in South Africa noted that HIV risk assessment and reduction were amongst key aspects of HIV that were not adequately discussed with clients during counselling. Instead, counselling mostly focused on details of HIV testing procedure and results interpretation [41]. Another recently published study conducted in South Africa observed that there was no improvement in HIV knowledge after counselling. This was attributed to quality assurance issues during the counselling process and to possible HCT inconsistencies [29]. Our study suggests that a questionnaire could be used to standardise counselling, whether it is used to collect risk behaviour data for surveillance or just as a reference tool to identify and address HIV risk factors during counselling. Other researchers have also shown that a tool used to collect information on risk behaviour could help in identifying risk of HIV acquisition [42, 43].

The limitations of this study are that it had a cross-sectional design, had fewer participants from the general HCT clinic, only enrolled participants who had negative rapid HIV tests who were screened for early HIV infection, and that it did not assess all the factors that can influence the risk of acquiring HIV such as educational level, socio-economic status and cultural or societal norms. There was also a lack of data on serial monogamous relationships for the majority of participants. Even though the study questionnaire was validated there could still be some inaccuracies in the behavioural data obtained from the participants owing to perceived sensitivity and stigma on certain questions in the context where questionnaires were administered by research assistants.

## Conclusions

Our data show that a large segment of sexually active South Africans in the Tshwane district have high risk exposure to HIV and that combination of risk factors play a role in HIV acquisition. The detection of newly diagnosed HIV infections in all study clinics reflects a wide distribution of individuals who are capable of sustaining HIV transmission in the setting where HIV risk behaviour is highly prevalent. This study also suggests that a questionnaire tool that captures data on HIV risk behaviour would be useful during HIV testing and counselling. This could ensure that there is a systematic way of identifying HIV risk factors during HCT such that counselling is optimised to identify and address the relevant risk factors for each individual. Systematic collection of HIV risk data during counselling would help to identify individuals who are eligible for biomedical interventions such as pre-exposure prophylaxis and circumcision. These data could also be used to inform relevant HIV prevention interventions that could be implemented at a community or population level. For instance, home-based HIV testing could be promoted in areas where most people are unaware of their partner’s HIV status as noted in this study. Continuous surveillance of HIV risk behaviour is needed to assess the risk of HIV acquisition in communities and the impact of HIV prevention measures.

## Supporting information

S1 Fig Questionnaire. A questionnaire tool that was used to collect demographic and HIV risk factors from the study participants. Codes (in red) were used to capture questionnaire data into the Excel spreadsheet. N/A—not applicable was used for participants who had no recent sexually transmitted diseases (number 12) and this also applied for parameters on number 18 and 19. Questionnaire parameters (numbers 5, 6, 8, 15 and 18) that were not applicable to the whole general or pregnant population group were not included in the group’s analysis but some of them were analysed separately.
(PDF)

S2 Fig Validation questionnaire. A questionnaire tool that was used to collect data for validation. This data was collected at a follow up visit.
(PDF)

S1 Table Validation questionnaire data obtained at a different time point. (DOCX)

S2 Table Some behavioural and biologic characteristics of participants with newly diagnosed HIV infection. (DOCX)
