# Supplementary material for: High risk exposure to HIV among sexually active individuals who tested negative on rapid HIV Tests in the Tshwane District of South Africa—The importance of behavioural prevention measures
Source: PLoS One. 2018 Feb 2;13(2):e0192357. doi: 10.1371/journal.pone.0192357 (PMC5796711; doi:10.1371/journal.pone.0192357)
Supplement: S1 Fig — A questionnaire tool that was used to collect demographic and HIV risk factors from the study participants. Codes (in red) were used to capture questionnaire data into the Excel spreadsheet. N/A—not applicable was used for participants who had no recent sexually transmitted diseases (number 12) and this also applied for parameters on number 18 and 19. Questionnaire parameters (numbers 5, 6, 8, 15 and 18) that were not applicable to the whole general or pregnant population group were not included in the group’s analysis but some of them were analysed separately. (PDF) [file pone.0192357.s001.pdf]

Have you been sexually active within the last 3 months? Yes ☐ No ☐

Participant number: ☐☐☐☐ **Date** .../.../....

Please answer the following questions (mark the appropriate box or use available space where needed).

1. **Age:** ☐☐ **Gender:** Male ☐ M Female ☐ F
2. **Race:** Black ☐ B White ☐ W Coloured ☐ C Indian ☐ I Other (specify): S________
3. **Marital status:** Single ☐ S Married ☐ M Divorced ☐ D
4. **Condom use:** Always ☐ A Sometimes ☐ S Never ☐ N
5. **Recent unprotected sex:** last week ☐ 1 2 weeks ago ☐ 2 3-4 weeks ago ☐ 3 2 months ago ☐ 4 3 months ago ☐ 5 None ☐ 0
6. **Reason for the last unprotected sex:** Condom burst ☐ B No use of condom ☐ N
7. **Is your partner HIV positive?** Yes ☐ Y No ☐ N Unknown status ☐ O
8. **Is your partner on ARVs?** Yes ☐ Y No ☐ N
9. **Alcohol use:** Daily ☐ D Every weekend ☐ W Occasionally ☐ C None ☐ 0
10. **Do you use drugs, e.g. cocaine?** Intravenous drugs ☐ Y Other: D________ No ☐ N
11. **Commercial sex worker:** Yes ☐ Y No ☐ N
12. **Recent STD?** Within the last 30 days ☐ 1 2 months ago ☐ 2 3 months ago ☐ 3 N/A = 0
13. **Sexual assault within the last month?** Yes ☐ Y No ☐ N
14. **Current number of sexual partners:** 1 ☐ 1 2 ☐ 2 3 - 5 ☐ 3 >5 ☐ 4
15. **Have you ever had sex with other men?** Yes ☐ Y No ☐ N N/A ☐ (for females) 0
16. **Male circumcision:** Yes ☐ Y No ☐ N N/A ☐ (for females) 0
17. **Are you a frequent traveller?** Yes ☐ Y No ☐ N
18. **Last time of travelling:** Within the last 30 days ☐ 1 2 months ago ☐ 2 3 months ago ☐ 3 N/A = 0
19. **Recent flu-like illness:** Within the last 30 days ☐ 1 2 months ago ☐ 2 3 months ago ☐ 3 N/A = 0
20. **Reason for HIV testing today?** VCT ☐ V **Circumcision** ☐ C Other P = pregnancy
