# Supplementary material for: High risk exposure to HIV among sexually active individuals who tested negative on rapid HIV Tests in the Tshwane District of South Africa—The importance of behavioural prevention measures
Source: PLoS One. 2018 Feb 2;13(2):e0192357. doi: 10.1371/journal.pone.0192357 (PMC5796711; doi:10.1371/journal.pone.0192357)
Supplement: S2 Fig — A questionnaire tool that was used to collect data for validation. This data was collected at a follow up visit. (PDF) [file pone.0192357.s002.pdf]

Have you been sexually active within the last 3 months? Yes ☐ Y No ☐ N

Participant number: ☐☐☐☐ **Date** …/…/….

Please answer the following questions (mark the appropriate box or use available space where needed).

1. **Age**: ☐☐ **Gender**: Male ☐ M Female ☐ F
2. **Race**: Black ☐ B White ☐ W Coloured ☐ C Indian ☐ I Other (specify): S ________
3. **Marital status**: Single ☐ S Married ☐ M Divorced ☐ D
4. **Condom use**: Always ☐ A Sometimes ☐ S Never ☐ N
5. **Have you talked about HIV status with your partner after your last HIV test?** Yes ☐ Y No ☐ N
6. **Is your partner HIV positive?** Yes ☐ Y No ☐ N Unknown status ☐ O
7. **Alcohol use**: Daily ☐ D Every weekend ☐ W Occasionally ☐ C None ☐ O
8. **Do you use drugs, e.g. cocaine?** Intravenous drugs ☐ Y Other: D ________ No ☐ N
9. **Have you changed sexual partners since participating in this study?** Yes ☐ Y No ☐ N
10. **Current number of sexual partners:** 1 ☐ 1 2 ☐ 2 3 - 5 ☐ 3 >5 ☐ 4
11. **Have you ever had sex with other men?** Yes ☐ Y No ☐ N N/A ☐ (for females) O
12. **Commercial sex worker:** Yes ☐ Y No ☐ N
