# Supplementary material for: High risk exposure to HIV among sexually active individuals who tested negative on rapid HIV Tests in the Tshwane District of South Africa—The importance of behavioural prevention measures
Source: PLoS One. 2018 Feb 2;13(2):e0192357. doi: 10.1371/journal.pone.0192357 (PMC5796711; doi:10.1371/journal.pone.0192357)
Supplement: S1 Table — (DOCX) [file pone.0192357.s003.docx]

Supplementary Table 1: Validation questionnaire data obtained at a different time point

| **FACTORS** | **SUB-GROUPS** | **PARTICIPANTS**  **% (n)** |
| --- | --- | --- |
|  |  |  |
| **Age** | 14 - 24 years | 34.6 (46) |
|  | 25 - 49 years | 65.4 (87) |
|  | 50+ years | -- |
|  |  |  |
| **Gender** | Males | -- |
|  | Females | 100 (133) |
|  |  |  |
| **Marital status** | Unmarried | 72.2 (96) |
|  | Married | 27.8 (37) |
|  |  |  |
| **Condom use** | Consistent | 1.5 (2) |
|  | Inconsistent | 50.8 (67) |
|  | No use | 57.1 (63) |
|  |  |  |
| **Partner’s HIV status** | Positive | 2.3 (3) |
|  | Negative | 40.6 (54) |
|  | Unknown | 63.0 (76) |
|  |  |  |
| **Number of current**  **sexual partners** | One | 100 (132) |
|  | Multiple | -- |
|  |  |  |
| **Alcohol use** | Yes | 0.7 (1) |
|  | No | 99.3 (132) |
|  |  |  |
| **Other drugs** | Yes | -- |
|  | No | 100 (133) |

-- = no data.
