# Supplementary material for: High risk exposure to HIV among sexually active individuals who tested negative on rapid HIV Tests in the Tshwane District of South Africa—The importance of behavioural prevention measures
Source: PLoS One. 2018 Feb 2;13(2):e0192357. doi: 10.1371/journal.pone.0192357 (PMC5796711; doi:10.1371/journal.pone.0192357)
Supplement: S2 Table — (DOCX) [file pone.0192357.s004.docx]

Supplementary Table 2. Some behavioural and biologic characteristics of participants with newly diagnosed HIV infection

|  | **Pt ID** | **Age** | **Gender** | **Race** | **Marital Status** | **Frequency of condom use** | **HIV status of the partner** | **Alcohol use** | **Marijuana use** | **Recent STI** | **Current number of sexual partners** | **Frequent travel to other countries** | **HIV viral loads (copies/ml)** |
| --- | --- | --- | --- | --- | --- | --- | --- | --- | --- | --- | --- | --- | --- |
| 1 | 9498 | 34 | F | B | S | No | Neg | No | No | No | ND | No | 5.0 x 10^5^ |
| 2 | 9218 | 30 | F | B | D | Incon | Neg | No | No | Yes | ND | Yes | 5.5 x 10^4^ |
| 3 | 9228 | 28 | M | B | S | No | Unk | Occ | Yes | Yes | 1 | Yes | 9.4 x 10^1^ |
| 4 | 2066 | 22 | F | B | S | Incon | Unk | Wknd | No | No | ND | No | 1.0 x 10^3^ |
| 5 | 8575 | 27 | F | B | S | Incon | Unk | No | No | No | 1 | No | 9.3 x 10^4^ |
| 6 | 7293 | 21 | F | B | S | Incon | Neg | Occ | No | No | 1 | No | 5.0 x 10^3^ |
| 7 | 5041 | 23 | M | B | S | Incon | Unk | Occ | No | No | 4 | No | 2.2 x 10^7^ |
| 8 | 8047 | 31 | M | B | M | No | Unk | Occ | No | No | 1 | No | 1.2 x 10^6^ |
| 9 | 9049 | 20 | F | B | S | Incon | Unk | No | No | No | 1 | No | 1.6 x 10^4^ |
| 10 | 6638 | 28 | F | B | S | Incon | Unk | No | No | No | 1 | No | 1.9 x 10^5^ |
| 11 | 261 | 33 | M | B | S | No | Pos | Wknd | No | No | 1 | No | 8.4 x 10^7^ |
| 12 | 6512 | 23 | F | B | S | Incon | Unk | No | No | No | 1 | No | 1.7 x 10^3^ |
| 13 | 6743 | 26 | F | B | S | Incon | Unk | No | No | No | 1 | No | 2.7 x 10^4^ |
| 14 | 6582 | 24 | F | B | S | No | Unk | No | No | No | 1 | No | 6.2 x 10^3^ |
| 15 | 6727 | 28 | F | B | S | Incon | Unk | No | No | No | 1 | No | 4.8 x 10^3^ |
| 16 | 6737 | 24 | F | B | S | Incon | Unk | No | No | No | 1 | No | 2.2 x 10^3^ |
| 17 | 7084 | 28 | F | B | S | No | Unk | No | No | No | 1 | No | 3.3 x 10^8^ |
| 18 | 2504 | 24 | F | B | S | Incon | Unk | No | No | No | 1 | No | 3.7 x 10^4^ |
| 19 | 3469 | 20 | F | B | S | No | Neg | No | No | No | 1 | No | 3.3 x 10^4^ |
| 20 | 5054 | 26 | F | B | S | No | Unk | No | No | No | 1 | No | 2.7 x 10^4^ |
| 21 | 5067 | 20 | F | B | S | No | Unk | No | No | No | 1 | No | 1.2 x 10^4^ |
| 22 | 9915 | 30 | F | B | M | No | Unk | No | No | No | 1 | No | 1.4 x 10^4^ |
| 23 | 4351 | 35 | F | B | S | No | Unk | No | No | No | 1 | No | 2.6 x 10^3^ |
| 24 | 639 | 30 | F | B | S | No | Unk | No | No | No | 1 | No | 6.5 x 10^3^ |
| 25 | 641 | 30 | F | B | S | Incon | Neg | No | No | No | 1 | No | 7.0 x 10^4^ |
| 26 | 7959 | 40 | F | B | S | Incon | Pos | No | No | No | 1 | No | 1.4 x 10^5^ |
| 27 | 8828 | 27 | F | B | M | Incon | Unk | No | No | No | 1 | No | 4.1 x 10^4^ |
| 28 | 2678 | 30 | F | B | M | No | Unk | No | No | No | 1 | No | 2.2 x 10^5^ |
| 29 | 9895 | 31 | F | B | M | No | Unk | No | No | No | 1 | No | 4.8 x 10^3^ |
| 30 | 9986 | 28 | F | B | S | No | Unk | No | No | No | 1 | No | 9.7 x 10^4^ |
| 31 | 843 | 21 | F | B | S | Incon | Pos | No | No | No | 1 | No | 2.9 x 10^4^ |
| 32 | 6990 | 26 | F | B | S | Incon | Unk | No | No | No | 1 | No | 1.7 x 10^4^ |
| 33 | 2340 | 22 | F | B | S | Incon | Unk | No | No | No | 1 | No | 1.4 x 10^4^ |
| 34 | 6709 | 28 | F | W | S | Incon | Unk | No | No | No | 1 | No | 5.3 x 10^1^ |
| 35 | 6748 | 28 | F | B | S | Incon | Unk | No | No | No | 1 | No | 9.3 x 10^2^ |
| 36 | 6671 | 35 | F | B | M | No | Unk | No | No | No | 1 | No | 1.4 x 10^4^ |
| 37 | 6380 | 25 | F | B | S | Incon | Unk | No | No | No | 1 | No | 1.1 x 10^4^ |
| 38 | 6557 | 38 | F | B | S | No | Unk | No | No | No | 1 | No | 6.1 x 10^2^ |
| 39 | 6565 | 28 | F | B | S | Incon | Unk | No | No | No | 1 | No | 5.6 x 10^3^ |
| 40 | 6509 | 36 | M | B | M | No | Pos | No | No | No | 1 | No | 1.0 x 10^5^ |
| 41 | 6596 | 31 | F | B | S | Incon | Unk | No | No | No | 1 | No | 3.8 x 10^3^ |
| 42 | 6640 | 37 | F | B | M | No | Unk | No | No | No | 1 | No | 3.0 x 10^3^ |
| 43 | 6649 | 32 | F | B | M | No | Unk | No | No | No | 1 | No | 2.1 x 10^4^ |
| 44 | 6738 | 28 | F | B | S | No | Unk | No | No | No | 1 | No | 1.5 x 10^5^ |
| 45 | 1067 | 19 | F | B | S | Con | Unk | Occ | No | No | 1 | No | 1.7 x 10^3^ |
| 46 | 921 | 44 | F | B | M | Incon | Neg | No | No | No | 1 | No | 9.7 x 10^3^ |
| 47 | 3869 | 32 | F | B | S | Incon | Unk | No | No | Yes | 1 | No | 2.1 x 10^5^ |
| 48 | 3912 | 27 | F | B | S | Incon | Neg | No | No | No | 1 | No | 3.2 x 10^4^ |
| 49 | 3920 | 20 | F | B | S | Incon | Neg | No | No | No | 1 | No | 6.6 x 10^4^ |
| 50 | 3880 | 30 | F | B | S | Incon | Neg | No | No | No | 1 | No | 7.5 x 10^3^ |
| 51 | 3935 | 33 | F | B | S | No | Unk | No | No | No | 1 | No | 2.4 x 10^5^ |
| 52 | 1117 | 37 | F | B | M | Incon | Unk | No | No | No | 1 | No | 1.5 x 10^2^ |
| 53 | 1121 | 27 | F | B | S | Incon | Unk | No | No | No | 1 | No | 8.0 x 10^4^ |
| 54 | 3474 | 21 | F | B | S | Incon | Neg | No | No | No | 1 | No | 1.6 x 10^4^ |
| 55 | 1475 | 32 | F | B | S | No | Neg | No | No | No | 1 | No | 4.4 x 10^4^ |
| 56 | 3387 | 37 | F | B | S | No | Neg | No | No | No | 1 | No | 7.9 x 10^4^ |
| 57 | 3253 | 28 | F | B | S | Incon | Neg | No | No | No | 1 | No | 8.9 x 10^4^ |
| 58 | 1692 | 18 | F | B | S | Incon | Unk | No | No | No | 1 | No | 3.9 x 10^2^ |
| 59 | 3606 | 24 | F | B | S | Incon | Unk | No | No | No | 1 | No | 1.5 x 10^4^ |
| 60 | 2866 | 35 | F | B | S | Incon | Unk | No | No | No | 1 | No | 3.3 x 10^3^ |
| 61 | 1213 | 36 | F | B | S | No | Neg | No | No | No | 1 | No | 3.2 x 10^4^ |

Pt ID – participant identity, B – black, W – white, S – single, M – male or married, D – divorced, Incon – inconsistent, Con – consistent, Neg – negative, Pos – positive, Unk – unknown, Occ – occasionally, Wknd – every weekend, STI – sexually transmitted infection. ND – no data.
